# The landscape of novel and complementary targets for immunotherapy: an analysis of gene expression in the tumor microenvironment

**DOI:** 10.18632/oncotarget.27027

**Published:** 2019-07-16

**Authors:** Stephen G. Gaffney, Elizabeth B. Perry, Ping-Min Chen, Andrew Greenstein, Susan M. Kaech, Jeffrey P. Townsend

**Affiliations:** ^1^ Department of Biostatistics, Yale University School of Public Health, New Haven, CT, USA; ^2^ Gilead Sciences, Foster City, CA, USA; ^3^ Department of Immunobiology, Yale University School of Medicine, New Haven, CT, USA

**Keywords:** immunotherapy, tumor microenvironment, T lymphocyte, cancer genomics, RNA-seq

## Abstract

**Background:** Immunotherapies targeting immune checkpoint proteins CTLA-4, PD-1, and PD-L1 have saved lives, but these therapies have only been effective in some patients. Patients positive for expression of immune checkpoint proteins in the tumor microenvironment show better response to immune checkpoint inhibitors. Consequently, knowledge of which genes are consistently expressed in lymphocytes within the tumor microenvironment can convey potentially effective and complementary new immunotherapy targets.

**Results:** We identified 54 genes that have higher co-expression with the pan T-cell marker *CD3E* than *CTLA4* or *PDCD1*. In a dataset of 26 patients who received anti-PD-1 therapy, we observed that co-expression between *CD3E* and *PDCD1* was higher among responders than non-responders, supporting our correlation-based approach.

**Conclusions:** The genes highlighted in these analyses, which include *CD6*, *TIGIT*, *CD96*, and *SLAMF6*, warrant further investigation of their therapeutic potential.

**Methods:** We analyzed and ranked genes that were co-expressed with the pan T-cell marker *CD3E* in 9,601 human tumors, spanning 31 cancer types. To further identify targets that may be complementary to existing PD-1 therapy, we examined and ranked genes with high *CD3E* co-expression and relatively low *PDCD1* co-expression.

## INTRODUCTION

The long-term effectiveness of broad-spectrum chemotherapies [1] and molecularly-targeted therapies [2] is mitigated by the evolutionary dynamics of tumor cells, wherein natural selection favors increasingly aggressive and drug-resistant clones. Therapeutic resistance presents one of the universal challenges in cancer treatment and can be attributed to the extensive genetic [3] and phenotypic [4] diversity present within tumor cell populations. Recent achievements in the field of immunotherapy [5–7] to treat advanced cancers have renewed optimism about harnessing the power of the adaptive immune system, and its ability to produce a nearly-unlimited diversity of antigen-recognizing receptors, to achieve lasting therapeutic results [8–10]. The promise of immunomodulatory approaches lies in the potential to confront one dynamic, diversity-rich system (the evolving tumor) with another (the adaptive immune system) [11–14]. Nevertheless, there are challenges to using the immune system to fight cancer: tumors appear to have a diversity of immune-evasion mechanisms that modulate the immune response [15–18]. As we learn more about the biomarkers that identify which immunotherapy approaches have promise for each patient [19–21], these challenges might be successfully addressed by developing a variety of approaches for immunomodulation in diverse patients and circumstances.

The goal of immunotherapy is typically to reverse tumor immune-evasion mechanisms and restore local immune response against cancer cells [22–25]. Cytotoxic T-lymphocyte associated protein 4 (CTLA-4), which represses early T-cell activation, was the first immune checkpoint receptor to be targeted therapeutically [26, 27]. Most recent immunotherapy studies focus on antibodies to block the programmed cell death protein 1 (PD-1) or its ligands (e.g. PD-L1), which have enabled breakthroughs in the treatment of melanoma, non-small cell lung cancer, and renal cell carcinoma [28–31]. These treatments can markedly improve patient survival, but only a minority of patients respond to therapy [32, 33]. Analysis of PD-1 blockade response data reveals increased response in patients with higher tumor PD-L1 expression, and higher PD-1 expression on the tumor infiltrating lymphocytes (TILs) [34]. These facts reaffirm the biological basis for immunotherapy: by reprogramming the suppressed TILs, it is possible to make tumors newly vulnerable to the immune system.

The development of an arsenal of approaches to modulate immune response is dependent on the identification and prioritization of targets that are likely to modulate immune activity in tumors in complementary ways to extant therapies [11–13]. To predict the best immunomodulatory treatment targets that would reverse the suppression of tumor-infiltrating T cells, an approach is needed that evaluates potential immunomodulatory interactions with T cells in the tumor microenvironment [35–37], analogous to approaches outside the realm of immunotherapy that identify targets based on functional associations of disease [38]. We reasoned that immunomodulatory targets would have to be relatively abundantly expressed in tumor-infiltrating T cells. Accordingly, we used mRNA expression of the pan T-cell marker *CD3E* as a metric for T-cell abundance within a tumor [12], and examined correlations between expression of *CD3E* and putative targets across 9,601 human tumors spanning 31 cancer types. We deemed genes whose expression is highly correlated with *CD3E*—and therefore are likely to be expressed in T cells within the tumor microenvironment—to be promising targets for therapy. We further reasoned that the most complementary targets would be those that are not co-expressed in common with *PDCD1* (the gene coding for PD-1), as they might function in individuals for whom anti-PD-1 therapy is insufficient or inviable. Therefore, we also examined the correlation of putative targets with *PDCD1* expression. We identified targets with high *CD3E* correlation and relatively low *PDCD1* correlation, suggestive of the presence of T cells with low PD-1 expression. These immune-related genes could potentially be new targets for therapy complementary to PD-1 blockade therapy. Lastly, we extended our analysis to consider complementarity to therapies directed at both PD-1 and CTLA-4.

## RESULTS

### T-cell associated genes exhibit consistent expression in tumor microenvironments across cancer types

**Figure 1:**
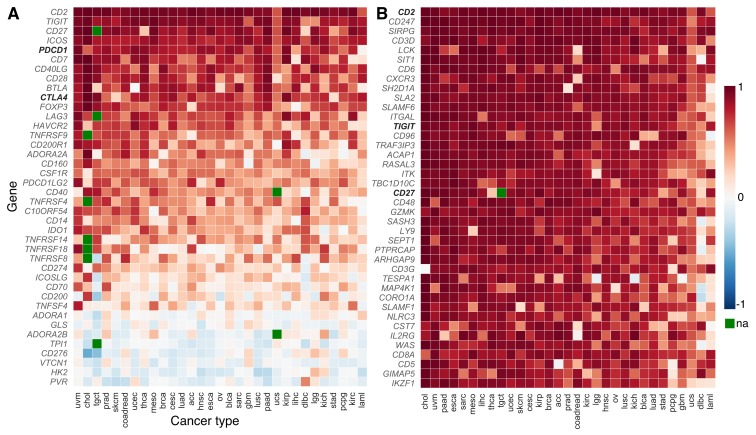
*CD3E* co-expression across cancer types (TCGA abbreviations; Supplementary Table 2). Among the (**A**) 40 candidate genes of the gene panel, several candidate genes show more correlated co-expression with the pan T-cell marker *CD3E* than successfully-targeted immune checkpoints like *PDCD1* and *CTLA4* (shown in bold). Candidate genes show remarkable consistency in their co-expression patterns across a variety of cancer types. Correlation coefficients in (**B**) a cancer exome-wide analysis reveal many genes that are highly-correlated with gene expression of pan T-cell marker *CD3E* in the tumor microenvironment. Higher correlation in expression than exhibited by *PDCD1* and *CTLA4* is observed in 54 and 102 genes, respectively. The gene *CD2* tops both lists. Only two other candidate genes (*TIGIT* and *CD27,* shown in bold) fall in the top-50 ranking in the exome-wide analysis (#13 and #20; Supplementary Figure 2). The color of each tile indicates correlation with *CD3E* expression, (blue, low to red, high; green: not assayed). The genes are ranked according to the median of correlation coefficients across cancer types. Candidate genes from the gene panel are shown in bold.

We generated a heat map to indicate the strength of correlation in expression between each gene in a 40-gene candidate panel and the pan T-cell marker *CD3E,* across 31 cancer types. Candidate genes were selected based on expert knowledge for their known functional relevance in T-cell function and their known effects on CD8 T-cell function. *CD3E* was selected as a T-cell marker because of its relatively reliable and broad expression in T cells. Analyses using genes encoding other components of CD3 gave similar results (Supplementary Figure 1). Differentiating between CD4^+^ and CD8^+^ T-cell markers did result in different rankings, consistent with evidence that these T-cell types play divergent roles in the tumor microenvironment [39–42]. We chose not to use a combination of genes in a signature-based analysis because we did not want our search for new targets to be biased by a predefined gene signature. Four of the candidate genes we examined showed more highly correlated expression with *CD3E* than was exhibited by the sometimes highly effective immunotherapy target and checkpoint inhibitor *PDCD1*. Another successful immunotherapy target, *CTLA4*, ranks eighth on the list of candidate genes in terms of how strongly correlated its co-expression is with the *CD3E* T-cell marker (Figure 1A). Genes tend to show highly consistent *CD3E* co-expression patterns across the 31 cancer types studied (Figure 1A). Additional potential gene targets are identified that have more highly correlated expression with *CD3E* than *PDCD1* (ranked #55) and *CTLA4* (ranked #103) when we expanded the list of studied genes from our 40-candidate list to all genes known to be expressed in cancer (Figure 1B, Supplementary Figure 2). Promisingly, these genes tend to have known immune regulatory functions (Table 1). The gene for the co-stimulatory cell-adhesion molecule CD2 ranks at the top of both the candidate and exome-wide lists.

**Table 1:** Genes with highest *CD3E* co-expression in exome-wide analysis

Rank^a^	Symbol	Pearson’s *r*	Gene description^b^	Panel?^c^
1	***CD2***	0.955	T-cell surface antigen CD2	✓
2	***CD247***	0.944	T-cell receptor T3 zeta chain	
3	***SIRPG***	0.942	signal-regulatory protein gamma; CD172g antigen	
4	***CD3D***	0.933	T-cell surface glycoprotein CD3 delta chain	
5	***LCK***	0.918	lymphocyte-specific protein tyrosine kinase	
6	***SIT1***	0.917	signaling threshold regulating transmembrane adaptor 1	
7	***CD6***	0.912	T-cell differentiation antigen CD6	
8	***CXCR3***	0.910	chemokine (C-X-C motif) receptor 3	
9	***SH2D1A***	0.907	T-cell signal transduction molecule SAP	
10	***SLA2***	0.904	Src-like-adaptor 2; modulator of antigen receptor signaling	
11	***SLAMF6***	0.902	SLAM family member 6; activating NK receptor	
12	***ITGAL***	0.897	antigen CD11A (p180), lymphocyte function-associated antigen 1	
13	***TIGIT***	0.896	T cell immunoreceptor with Ig and ITIM domains	✓
14	***CD96***	0.896	T cell activation, increased late expression	
15	***TRAF3IP3***	0.892	TRAF3-interacting JNK-activating modulator	
16	***ACAP1***	0.882	ArfGAP with coiled-coil, ankyrin repeat and PH domains 1	
17	***RASAL3***	0.882	RAS protein activator like 3	
18	***ITK***	0.879	interleukin-2-inducible T cell kinase	
19	***TBC1D10C***	0.879	carabin; TBC1 domain family, member 10C; RAS signaling inhibitor	
20	***CD27***	0.877	T-cell activation antigen CD27	✓
21	***CD48***	0.872	CD48 antigen (B-cell membrane protein)	
22	***GZMK***	0.870	granzyme K (granzyme 3; tryptase II)	
23	***SASH3***	0.865	SAM and SH3 domain containing 3	
24	***LY9***	0.865	lymphocyte antigen 9	
25	***SEPT1***	0.865	septin 1; serologically defined breast cancer antigen NY-BR-24	
26	***PTPRCAP***	0.862	protein tyrosine phosphatase, receptor type, C-associated protein	
27	***ARHGAP9***	0.858	Rho GTPase activating protein 9	
28	***CD3G***	0.857	T-cell receptor T3 gamma chain	
29	***TESPA1***	0.856	thymocyte expressed, positive selection associated 1	
30	***MAP4K1***	0.854	MAPK/ERK kinase kinase kinase 1	
31	***CORO1A***	0.842	coronin, actin binding protein, 1A	
32	***SLAMF1***	0.841	signaling lymphocytic activation molecule family member 1	
33	***NLRC3***	0.839	NLR family, CARD domain containing 3	
34	***CST7***	0.838	cystatin F; leukocystatin	
35	***IL2RG***	0.838	interleukin 2 receptor, gamma	
36	***WAS***	0.837	Wiskott-Aldrich syndrome	
37	***CD8A***	0.834	T-cell surface glycoprotein CD8 alpha chain	
38	***CD5***	0.829	CD5 antigen (p56-62)	
39	***GIMAP5***	0.828	immunity-associated nucleotide 5 protein	
40	***IKZF1***	0.828	IKAROS family zinc finger 1 (Ikaros)	
41	***ZAP70***	0.828	zeta chain of T-cell receptor associated protein kinase 70	
42	***KLRK1***	0.827	killer cell lectin like receptor K1	
43	***CCL5***	0.827	C-C motif chemokine ligand 5	
44	***GPR171***	0.826	G protein-coupled receptor 171	
45	***PVRIG***	0.825	transmembrane protein PVRIG; CD112 receptor	
46	***PTPN7***	0.824	protein tyrosine phosphatase, non-receptor type 7	
47	***CXCR6***	0.823	C-X-C motif chemokine receptor 6	
48	***EVI2B***	0.823	ecotropic viral integration site 2B	
49	***CCR5***	0.819	C-C motif chemokine receptor 5	
50	***GZMA***	0.815	granzyme A; Cytotoxic T-lymphocyte-associated serine esterase-3	
51	***ICOS***	0.815	inducible T-cell co-stimulator	✓
52	***GRAP2***	0.813	GRB2-related adaptor protein 2	
53	***PTPRC***	0.808	CD45; protein tyrosine phosphatase, receptor type, C	
54	***GIMAP4***	0.806	GTPase, immunity-associated protein 4	
55	***PDCD1***	0.806	programmed cell death 1	✓
103	***CTLA4***	0.727	cytotoxic T-lymphocyte associated protein 4	✓

^a^Genes are ranked according to median *CD3E* co-expression across cancer types.

^b^Gene descriptions are taken from the NCBI Gene database.

^c^Present in the 40-gene panel if indicated by a check mark.

### Candidate gene targets to complement anti-PD1 therapies

**Figure 2:**
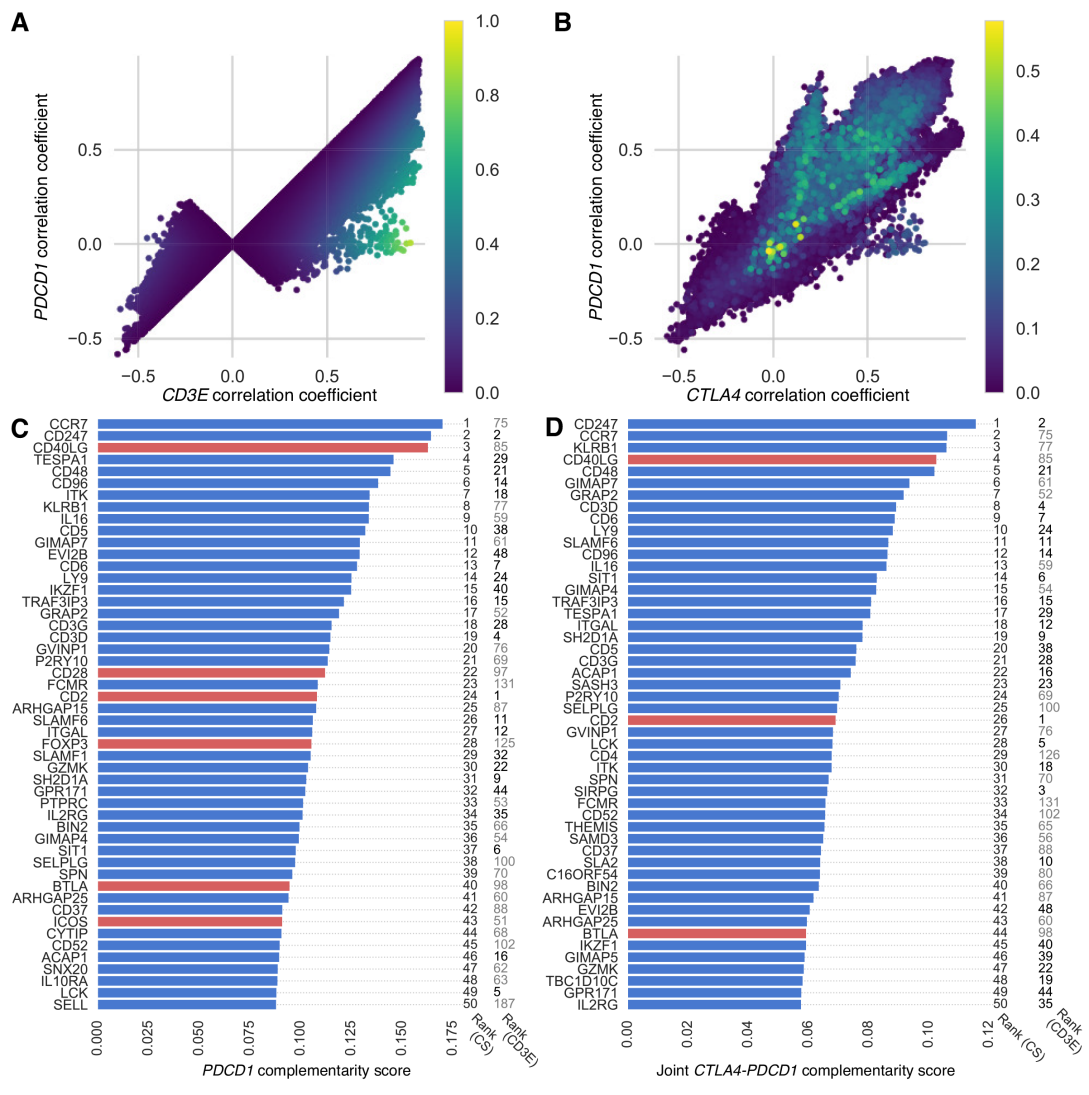
*PDCD1*-complementarity and joint *CTLA4-PDCD1* complementarity scores of exome-wide gene list. Scatter plots show correlations for each target gene, with results from each cancer type superimposed and colored (blue, low to yellow, high) according to (**A**) *PDCD1*-complementarity score, which is high when expression of *CD3E* and *PDCD1* are respectively correlated and uncorrelated with the target gene—genes with the highest *PDCD1*-complementarity scores are highly correlated with *CD3E* (approaching one on the *x*-axis) and less correlated with *PDCD1* (approaching zero on the *y*-axis); and (**B**) joint *CTLA4*-*PDCD1* complementarity score (including only scores above zero), which is high when expression of *CD3E* is correlated with the target gene and expression of *PDCD1* and *CTLA4* are uncorrelated with the target gene. Genes with the highest joint complementarity scores have close to zero correlation in expression with both *CTLA4* and *PDCD1*. Bar plots show the top 50 genes ranked according to (**C**) their median *PDCD1*-complementarity score and (**D**) their joint *CTLA4*-*PDCD1* complementarity score across cancer types. Top candidate genes (red bars) and top genes from the cancer exome (blue bars) are shown. To the right of each bar is its ranking based on complementarity score (CS) and *CD3E* co-expression (CD3E). Ranks up to 50 are in boldface.

We re-ranked the 40-candidate gene panel and the exome-wide gene lists, using a “*PDCD1*-complementarity score” that simultaneously considers the strength of correlation between the gene target and *CD3E* (as above), and the strength of correlation in gene expression between the target and *PDCD1*. This re-ranking provided a list of genes that could potentially serve not only as effective therapeutic targets in general, but as complementary therapies to anti-PD-1 therapy, in that they might be especially effective for patients whose tumors do not respond to anti-PD-1 therapy. Genes with the highest *PDCD1*-complementarity score were highly correlated in expression with *CD3E* and less correlated with *PDCD1* expression (Figure 2A, Supplementary Figure 3). When taking complementarity to *PDCD1* into account, the C-C chemokine receptor type 7 gene *CCR7* tops the list, and *CD2* ranks #24. Among genes in our 40-candidate panel, *CD40LG*, *CD2*, and *BTLA* remain highly complementary in our joint analysis of complementarity to therapies directed at *PDCD1* and to *CTLA4* (Figure 2B–2D, Supplementary Figure 4).

GSEA pathway enrichment analysis of the exome-wide gene list ranked by *CD3E* co-expression revealed significant enrichment of immune-related pathways (Table 2; Supplementary Table 1). The top 50 pathways all have FDR *Q* value below 1 × 10^−5^, ranked by normalized enrichment scores in the range 2.06–2.38. The top-ranked pathway is TCR signaling in naïve CD4^+^ T cells (curated by the Pathway Interaction Database, Supplementary Figure 5). Other pathways of interest include the CD8 TCR pathway, numerous immunoregulatory interactions, natural killer cell mediated cytotoxicity, and costimulation by the CD28 family; PD-1 has recently been demonstrated to exert its primary effect via regulation of CD28 [43–45].

**Table 2:** The top ten enriched pathways from GSEA

Rank	Pathway name	NES^a^
1	TCR pathway (PID)	2.383
2	CD8 TCR pathway (PID)	2.366
3	Immunoregulatory interactions between lymphoid & non-lymphoid cells (REACTOME)	2.360
4	Hematopoietic cell lineage (KEGG)	2.315
5	Class A1 rhodopsin like receptors (REACTOME)	2.302
6	Interferon gamma signaling (REACTOME)	2.302
7	Natural killer cell mediated cytotoxicity (KEGG)	2.300
8	Cell adhesion molecules cams (KEGG)	2.280
9	Cytokine cytokine receptor interaction (KEGG)	2.259
10	Costimulation by the CD28 family (REACTOME)	2.251

^a^Normalized Enrichment Score. All pathways listed have a false discovery rate *Q* < 1 × 10^−5^.

### Patients with response to anti-PD1 therapy have higher correlation between PDCD1 and CD3E expression than non-responders

**Figure 3:**
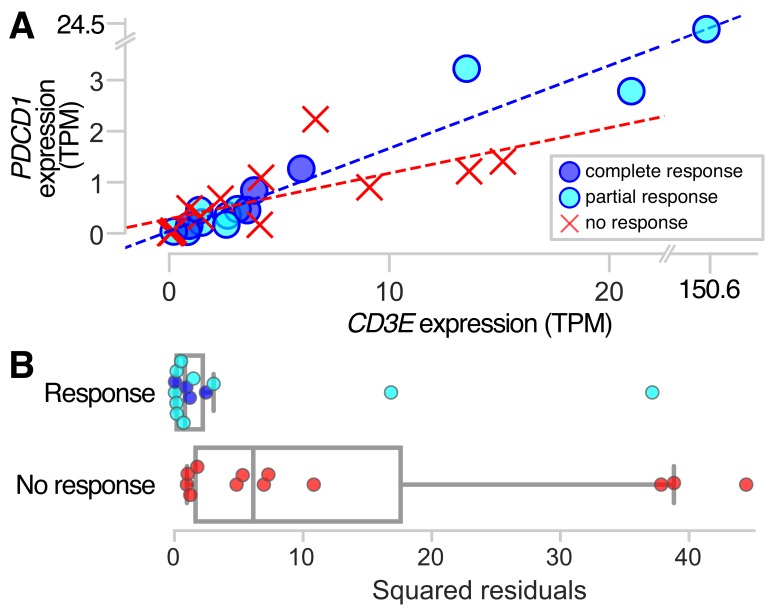
*PDCD1* and *CD3E* expression in responders and non-responders to anti-PD-1 therapy. Pretreatment samples from metastatic melanoma patients who ultimately responded to anti-PD-1 therapy show more highly correlated co-expression between *PDCD1* and the pan T-cell marker *CD3E,* as indicated by (**A**) a linear regression of the expression values for responders with a coefficient of determination *R*^2^ = 0.997, compared to *R*^2^ = 0.473 for non-responders; and (**B**) squared residuals from the linear regression of *CD3E* and *PDCD1* that are significantly lower for responders than non-responders (*P* = 0.003).

To examine how the relationship between *CD3E* expression and *PDCD1* expression influences response to anti-PD-1 therapy, we analyzed RNA-Seq transcriptome data from pretreatment samples of 26 metastatic melanoma patients who received anti-PD-1 therapy [82]. Patients had been categorized as ‘responders’ (*n* = 10 who experienced partial response, *n* = 4 who experienced complete response) and ‘non-responders’ (*n* = 12 who experienced progressive disease). Comparing *CD3E* and *PDCD1* expression among responders and non-responders, we find higher correlation among responders than non-responders (Figure 3A). Among the ‘responder’ patients, the Pearson’s correlation coefficient for transcript per million (TPM) expression values for *CD3E* and *PDCD1* is 0.998, while ‘non-responders’ have a lower correlation coefficient of 0.688. A linear regression of these expression values for responders yields an *R*^2^ statistic of 0.997, compared to a value of 0.473 for non-responders, indicating that a linear model has over twice the explanatory power for responders than non-responders. Squared residuals from the linear regressions are significantly lower for responders than non-responders (Figure 3B; *P* = 0.003, Mann–Whitney *U* test). One patient in the cohort, a responder, was an outlier with very high *PDCD1* and *CD3E* expression values (Figure 3A). When this patient is excluded from the analysis, the pattern holds. Analysis of the responder cohort excluding the outlier yields a *CD3E-PDCD1* correlation coefficient of 0.931, *R*^2^ of 0.868, and squared residuals from the linear regression remain significantly lower than non-responders; *P* = 0.005. These findings support our hypothesis that the immune regulatory genes we have discovered with highly correlated expression with *CD3E* may be promising candidates for new targeted-therapies.

## DISCUSSION

We examined a 40-gene panel of candidate immune regulators and an unbiased list of 12,082 expressed genes in cancer to find genes consistently co-expressed with the T-cell marker *CD3E* in the tumor microenvironment. We found that expression patterns were remarkably consistent across the 31 cancer types analyzed, and that the top genes were highly enriched within immune-related pathways, indicating that tumor-infiltrating T cells may have universal characteristics that could be targeted effectively in multiple cancers. We also identified T-cell associated genes whose expression patterns do not correlate highly with *PCDC1* that might be targeted for development of complementary therapies to anti-PD-1, or for patients who do not respond to anti-PD-1 therapy. When we examined anti-PD-1 responders and non-responders in metastatic melanoma, we found that responders had a significantly higher correlation between *CD3E* and *PDCD1* than did non-responders, which supports the hypothesis that correlation in expression with *CD3E* in the tumor microenvironment can be a useful criterion for identifying new therapeutic targets with potential for therapeutic response. It has been shown that although PD-L1 expression in tumor biopsies does appear to predict response to anti-PD-1 therapies, many tumors predicted as PD-L1 positive do not respond, while some responses occur in PD-L1-negative tumors [46–51]. Our results are similar in that we find that some patients with high *CD3E-PDCD1* correlation are not responders, whereas others with lower correlation do respond to anti-PD1 therapy. Also, when we performed a similar analysis on response to immune checkpoint therapies among patients with clear cell renal carcinoma, we did not find significant differences in *CD3E-PDCD1* correlation in responders and non-responders [52]. More detailed expression analyses of responders and non-responders in additional cancer types will help to shed light on additional genetic and tumor microenvironmental factors that influence response to new and existing and therapies [52, 53].

As a purely correlation-based approach, our exome-wide expression analysis is very coarse-grained, and does not incorporate multi-omic pharmacogenomic data [54] or the specific molecular biology of the genes identified. Extensive molecular biological research on their functional and structural properties is required to assess their viability as immunomodulatory targets [55, 56]. However, the guidance provided by our approach complements extant molecular biological investigation, highlighting well-studied genes that deserve continued attention as well as pointing out genes whose molecular biology is less well known, but which could be important for future research. While a subset of these genes will lack exploitable properties or would produce undesirable outcomes if they were targeted, it is also likely that novel and complementary targets that do have high potential are highlighted by our analysis. Furthermore, the consistency of the results of our analysis across cancer types inspires confidence that successful targeting of the genes could yield a high breadth of therapeutic applicability either alone or in combination with other therapies.

Among the more well-studied genes with potential for targeting, some have been previously identified by other approaches. CD6, a known T-cell co-stimulatory molecule, was identified in the exome-wide analysis as being co-expressed with *CD3E*. The binding of CD6 to CD166 (also called *ALCAM*: activated leukocyte-cell adhesion molecule) enables formation of a functional immune synapse. Indeed, an antibody that particularly antagonizes the function of CD6—Itolizumab—is under investigation for use as an anti-inflammatory in psoriasis patients [57, 58]. This antibody presents a possibility of stabilizing (i.e. agonizing) the CD6:CD166 interaction in the immune synapse so as to stimulate effector T-cell function. Of course, the systemic consequences of such an agent might outweigh the local benefits of a productive anti-tumor immune response.

Other well-studied targets are encoded by *TIGIT* and *CD96* [59, 60], which were similarly highly co-expressed with *CD3E*, and are known to inhibit effector T-cell activation. TIGIT and CD96 compete against the stimulatory receptor CD226 for shared ligands (i.e. CD155) and thus suppress CD226 activation [61]. TIGIT can suppress effector T-cell function by directly suppressing CD226 in *cis* or suppress APC function by signaling through CD155 in *trans* [62]. TIGIT and CD96 both suppress natural killer (NK) cell function as well [63, 64], and animal models of CD96 knockout mice revealed hyperinflammatory status with increased IFN-γ production in NK cells [64]. These mice are also resistant to a experimental lung metastasis model, suggesting a potential therapeutic role of CD96 blockade in cancer treatment [65]. TIGIT is known to additionally result in immunosuppression mediated by T regulatory cells (via secretion of IL-10 and TGF-β) [66]. Immunoregulatory function of TIGIT was shown to occur as a consequence of engagement with CD155 on dendritic cells, which results in increase IL-10 production, suppressing the effector T cells while promoting regulatory T cells [62]. Given the known functions of these receptors, the *CD3E* co-expression data reported here provides additional rationale for the development of selective antagonists and context for potential therapeutic application.

Expression of *SLAMF6* (also called *Ly108*)—a CD2 family member that plays a critical role in NK-cell development and activation—is also correlated with *CD3E* expression. SLAMF6 is known to be expressed on T cells, and its co-stimulation was shown to drive naïve CD4^+^ T cells toward a Th1 phenotype, inducing IFN-γ production [67]. SLAMF6 educates NK cells by forming homodimers at a synapse between cells. This homodimerization reduces NK-cell activity toward hematopoietic (i.e. SLAMF6^+^) cells while enhancing activity toward non-hematopoietic (SLAMF6^−^) tumor cells [68]. During development, SLAMF6 also reduces NK-cell differentiation and proliferation [69, 70]. Antibodies targeting SLAMF6 have demonstrated efficacy in mouse oncology models [71], underscoring the therapeutic potential of targeting this receptor and highlighting the potential of NK cells to play a critical role in anti-tumor immunity.

Additional immunotherapy-relevant patterns emerge when the genes that are highly ranked in our analyses are considered in aggregate. For instance, the high rank of the NK-cell mediated cytotoxicity pathway in the GSEA analysis, as well as pathways that include NK-cell associated proteins (e.g. *SLAMF6*), could reflect the prevalence of MHC loss or reduction in T-cell rich tumors. Loss or reduction of MHC is an emerging mechanism of immune evasion by tumor cells [72–74]. MHC loss or reduction would simultaneously be expected to reduce presentation of tumor-associated antigens to the T cells and, conversely, to make the tumor cells more susceptible to NK-cell targeting. This implication of susceptibility indicates potential value in stimulating NK-cell activation toward tumor cells that have lost MHC expression. Our analysis does not partition gene expression associations among the various T-cell populations (e.g. conventional ab T cells, gd T cells or NK T cells), but future work could examine correlations within partitions.

Our analysis informs immunomodulatory target selection for tumors with infiltrating T cells but low *PDCD1* expression. Other target-identification efforts could be aimed at recruiting T cells to the tumor or eliminating physical barriers (e.g. extracellular matrix) that limit the ability of effector T cells to exert cytotoxic effects on the tumor cell directly. It is possible that both approaches will be required in concert to ultimately drive the desired response in patients. For example, the remarkable success of chimeric-antigen receptor T-cell therapy (CAR-T therapy) in leukemias has been contrasted against more limited effects in solid tumors and some lymphomas. Targets identified in this report could also be relevant to the engineering of T cells to target solid tumors. Limited efficacy of CAR-T therapy in solid tumors might arise due to local signals that suppress cytotoxic effects upon arrival of the engineered T cells to the tumor microenvironment. The targets and pathways identified in this report might provide guidance regarding engineering approaches (i.e. Cas9-mediated knockouts or expression of decoy receptors) that could be applied where traditional antibody and small-molecule inhibitors are not feasible.

Immunotherapy approaches have produced some remarkable therapeutic successes, yet there is much uncharted territory left to explore. By identifying genes expressed in common with T-cell markers within the tumor microenvironments of 9,601 patients and 31 types of cancer, our work helps to map the boundaries of this landscape, narrowing the list of gene candidates for new therapeutic targets. Continued research will map out the immune composition and dynamic nature of the tumor microenvironment—providing opportunities to identify novel and complementary targets, and expanding the efficacy of therapeutics and the breadth of patients who benefit.

## MATERIALS AND METHODS

### Detecting T-cell associated gene expression in the tumor microenvironment

RNA-Seq V2 data from 31 tumor types (including 9601 tumor samples total) were obtained from The Cancer Genome Atlas (TCGA) using the open platform cBio Cancer Genomics Portal [75, 76] in the form of RSEM *z*-scores. Though available from this source, we excluded thymoma from our analysis because tumors of the thymus (where T cells mature) are known to directly alter the T-cell composition [77]. To find candidate genes expressed by T cells in the tumor microenvironment, we generated lists of genes for each cancer type, ranked by the strength of their correlation with *CD3E* expression, a pan T-cell marker [78]. Pearson product-moment correlation coefficients were calculated using the *corrcoef* method of Python’s numpy library, applied to all tumors with expression values for both *CD3E* and a given gene of interest. A weak correlation between a candidate gene and the T-cell marker could indicate that not all T cells in the tumor express the gene, that there is variability in the amount of expression among T cells in the tumor, and/or that cells other than T cells in the tumor are expressing the gene. Each of these possibilities could have implications for the effectiveness of T-cell-based targeted therapy, and all support the conclusion that targets with weaker correlations may be less successful than targets with stronger correlations.

We first performed this analysis on a candidate gene panel of 40 known immune-associated genes, and then we extended the analysis to an unbiased ranking of the cancer exome (12082 genes found to be expressed in cancer) [79]. The candidate gene panel included costimulatory genes (*CD2*, *CD27*, *CD28*, *TNFRSF8*, *CD40*, *CD40LG*, *CD70*, *ICOS*, *ICOSLG*, *TNFRSF4*, *TNFSF4*, *TNFRSF9*), putative T-cell inhibitory genes (*CTLA4*, *PDCD1*, *CD274*, *PDCD1LG2*, *VSIR*, *CD160*, *TNFRSF14*, *CD200*, *CD200R1*, *TIGIT*, *CD276*, *VTCN1, BTLA*, *LAG3*, *HAVCR2*), regulatory markers (*FOXP3*, *TNFRSF18*), and metabolic checkpoints (*ADORA1*, *ADORA2A*, *ADORA2B*, *HK2*, *GLS*, *IDO1*, *TPI1*). We also included several myeloid cell-related immune checkpoints (*CD14*, *CSF1R*, *KIR2DL1*, *PVR*), based on their involvement in the complex cell-cell interaction within the tumor microenvironment.

### Identifying candidate gene targets to complement anti-PD1 therapies

We developed a scoring metric to rank candidate genes according to their potential usefulness as alternative or complementary targets to *PDCD1*, an immune checkpoint gene that has already been successfully targeted in several cancer types. This metric, which we term ‘*PDCD1* complementarity score’, takes into account each gene’s strength of correlation with *CD3E* expression (to indicate consistent expression by T cells in the tumor microenvironment), and also minimizes the overlap in expression pattern with *PDCD1*. The goal of this analysis is to identify genes that will potentially be useful as targets for combined immunotherapy and/or for the subset of patients who do not respond to anti-PD-1 therapy. The promise of a target gene can be quantified with a function of the co-expressions of two pairs of genes: the target gene and *CD3E*; and the target gene and *PDCD1*, by the score

$$|\rho_{TC}|(|\rho_{TC}|-|\rho_{TP}|),$$

where co-expression is captured by the absolute value of their Pearson correlation coefficients, denoted |*ρ_TC_*| and |*ρ_TP_*|, respectively. While negative co-expression with *CD3E* could in principle give a high complementarity score, no high-scoring genes (e.g. in the top 600 of our ranked list) exhibited negative co-expression with *CD3E*.

### Identifying candidate gene targets to complement anti-PD1 and anti-CTLA-4 therapies

To identify genes that could be complementary to *CTLA4* as well as *PDCD1*, we extended the complementarity score to include co-expression with *CTLA4*. The joint complementarity score for a candidate gene was quantified as

$$|\rho_{TC}|\;\min\{|\rho_{TC}|-|\rho_{TP}|,|\rho_{TC}|-|\rho_{TA}|\},$$

where |*ρ_TP_*| is the absolute value of the Pearson correlation coefficient for the target expression and *CTLA4* expression. Genes with a high joint complementarity score have a high co-expression with *CD3E* and relatively low co-expression with both *PDCD1* and *CTLA4*.

### Gene set enrichment analysis

To identify overrepresented pathways among genes with high *CD3E* co-expression, we used the gene- set enrichment software GSEA (v3.0) from the Broad Institute [80, 81], using the ‘GSEAPreranked’ algorithm. Our input rank file consisted of the exome-wide gene set and each gene’s corresponding median co-expression value across the 31 cancer types. We tested against the ‘Canonical Pathways’ gene set (v6.1), selecting the weighted algorithm with default parameters. Results were compared using Normalized Enrichment Score (NES) and false discovery rate (FDR).

### Linking anti-PD1 therapy response to strength of correlation between CD3E and PD-1 expression in the tumor microenvironment

We accessed RNA-Seq transcriptome data from pretreatment samples of 26 metastatic melanoma patients who received anti-PD1 therapy, and that had been categorized as ”responders” (*n* = 10 who experienced partial response, plus *n* = 4 who experienced complete response) and ”non-responders” (*n* = 12 who experienced progressive disease) according to irRECIST criteria [82]. We looked for differences between responders and non-responders in terms of the strength of correlation between *CD3E* expression and *PDCD1* expression. Because patients whose tumor-infiltrating T cells consistently express *PDCD1* are more likely to respond to anti-PD1 therapy [5, 83, 84], we hypothesized a stronger correlation for responders than non-responders. Following the same logic, we also hypothesized that genes that are candidates for future tumor-infiltrating T-cell therapies will show high correlation in expression with *CD3E* in the tumor microenvironment. To assess differences in the *CD3E*-*PDCD1* correlation between responders and non-responders, we calculated Pearson’s correlation coefficients as described above, and performed linear regressions using the ols function of Python’s statsmodels library. We quantified linear model fits by comparing their coefficients of determination (*R*^2^), and evaluated the statistical significance of the best fits using the Mann–Whitney *U* test.

## SUPPLEMENTARY MATERIALS



## Abbreviations

CTLA-4: Cytotoxic T-lymphocyte associated protein 4
PD-1: Programmed cell death protein 1
PD-L1: Programmed cell death protein 1 ligand
TILs: Tumor infiltrating lymphocytes
TCGA: The Cancer Genome Atlas
GSEA: Gene set enrichment analysis
NES: Normalized Enrichment Score
FDR: False discovery rate
TPM: Transcript per million
NK: Natural killer
CAR-T therapy: Chimeric-antigen receptor T-cell therapy
