# Supplementary material for: The landscape of novel and complementary targets for immunotherapy: an analysis of gene expression in the tumor microenvironment
Source: Oncotarget. 2019 Jul 16;10(44):4532–45. doi: 10.18632/oncotarget.27027 (PMC6642048; doi:10.18632/oncotarget.27027)
Supplement: Supplementary file 1 [file oncotarget-10-4532-s001.pdf]

The landscape of novel and complementary targets for immunotherapy: an analysis of gene expression in the tumor microenvironment

SUPPLEMENTARY MATERIALS

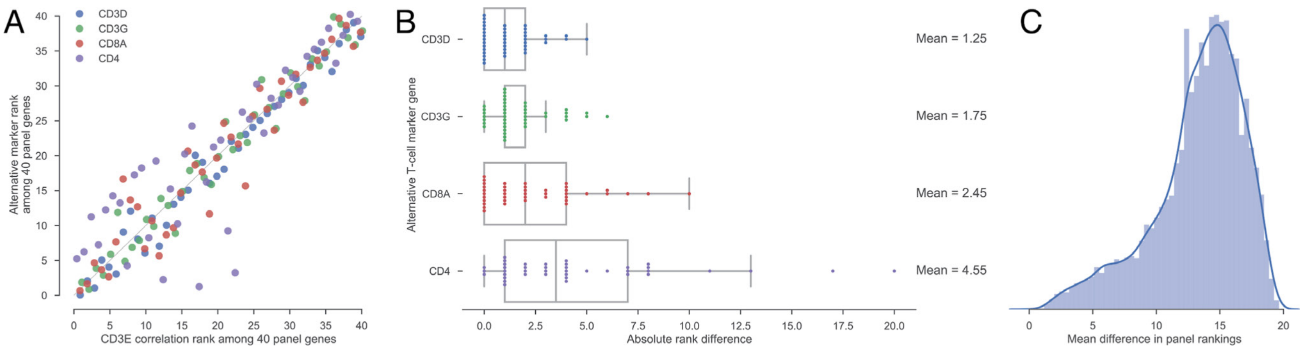

Supplementary Figure 1: Comparison of panel gene co-expression rankings when using alternative T-cell markers. Genes *CD3D, CD3G, CD8A*, and *CD4* were examined as alternative T-cell markers to *CD3E*. We calculated their co-expression with each of the 40 immunomodulatory panel genes, taking median coexpression across cancer projects, for comparison with results for *CD3E*. (**A**) A scatter plot shows ranks of the 40 panel genes, when sorted in descending order of co-expression with *CD3E* (x-axis) or an alternative marker (y-axis). Distance from the diagonal provides a measure of dissimilarity in rankings. This dissimilarity is quantified in (**B**) a box plot showing absolute values of differences in rankings. *CD3D* rankings have the greatest similarity to *CD3E* rankings (mean rank difference = 1.25), while *CD4* rankings have the least similarity (mean rank difference = 4.55). (**C**) A histogram showing the distribution of mean rank differences (versus *CD3E* rankings) across the exome (12076 genes). Using this distribution to map mean rank differences to percentiles, alternative markers *CD3D, CD3G, CD8A*, and *CD4* are at 0.03%, 0.18%, 0.61%, and 3% respectively, indicating a high degree of similarity to *CD3E*.

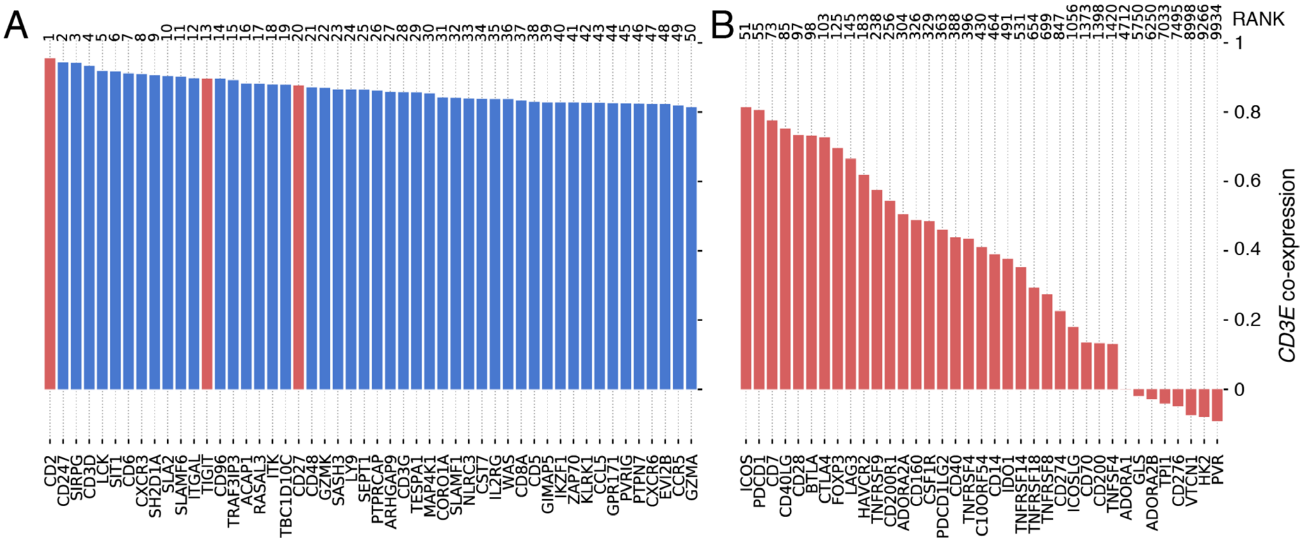

Supplementary Figure 2: Genes ordered by Pearson correlation coefficient for co-expression with *CD3E*, including (**A**) the top 50 genes (red bars: in the 40-candidate immunomodulatory gene panel, blue bars: in our cancer exome-wide gene list, but not in our original 40-candidate immunomodulatory gene panel), and (**B**) remaining genes from the 40-candidate immunomodulatory gene panel.

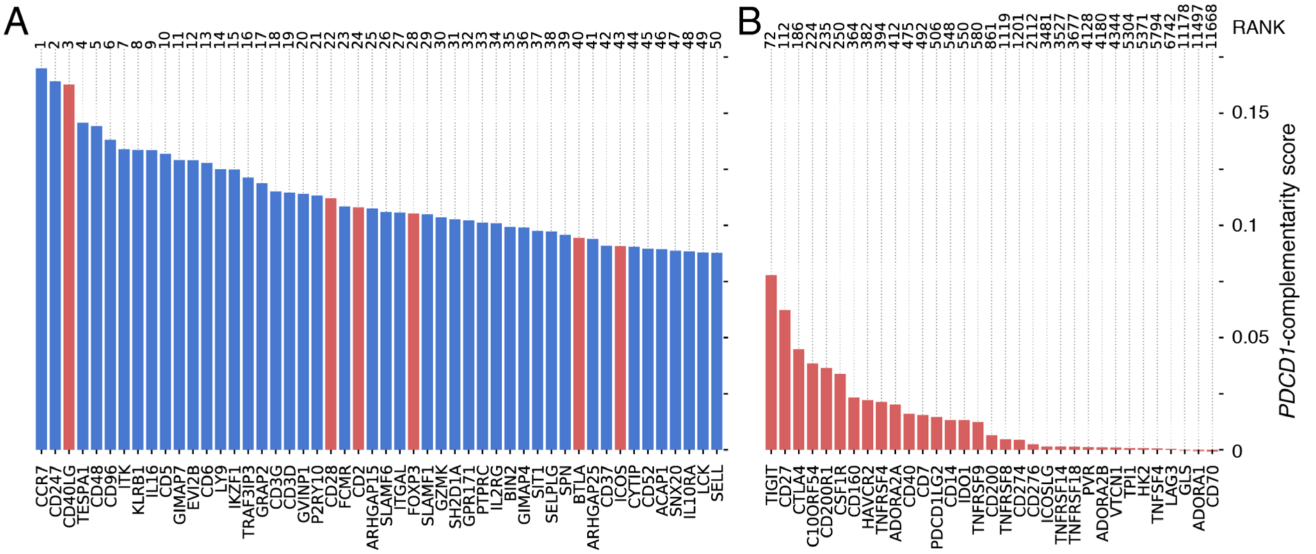

Supplementary Figure 3: Genes ordered by PDCD1-complementarity score, including (**A**) the top 50 genes (red bars: in the 40-candidate immunomodulatory gene panel, blue bars: in our cancer exome-wide gene list, but not in our original 40-candidate immunomodulatory gene panel), and (**B**) remaining genes from the 40-candidate immunomodulatory gene panel.

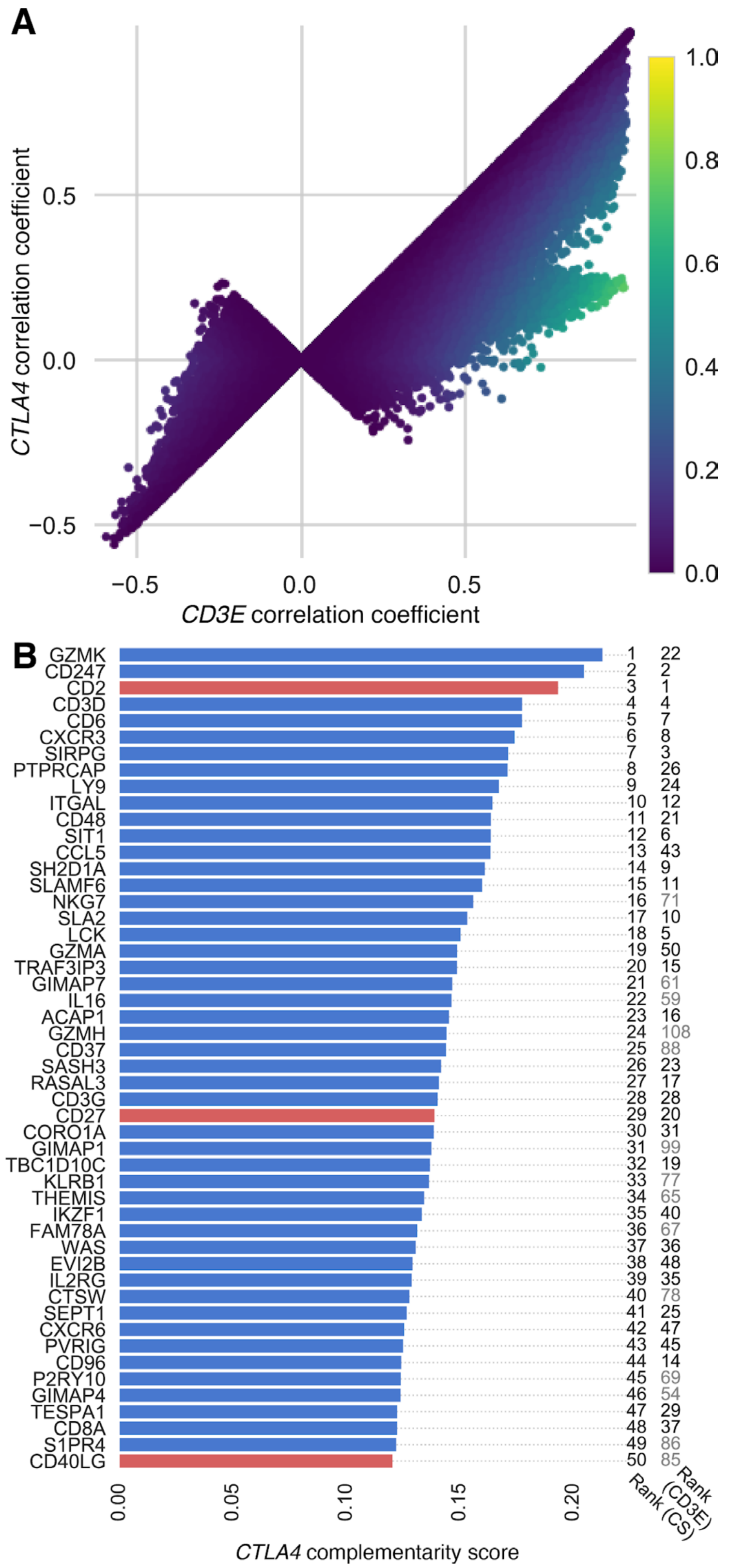

Supplementary Figure 4: *CTLA4*-complementarity scores for the cancer exome-wide gene list, displayed as (**A**) a scatter plot illustrating *CTLA4* and *CD3E* correlations for each target gene. Results from each cancer type are superimposed and colored according to *CTLA4*-complementarity score, which is high when expression of *CD3E* is correlated with the target gene and *CTLA4* is uncorrelated with the target gene. (**B**) A bar plot (red bars: in the 40-candidate immunomodulatory gene panel, blue bars: in our cancer exome-wide gene list, but not in our original 40-candidate immunomodulatory gene panel) shows the top 50 genes from our cancer exome-wide analysis, ranked according to their median *CTLA4*-complementarity score across cancer types. To the right of each bar is its ranking based on complementarity score (CS) and *CD3E* co-expression (*CD3E*; typeface is plain when *CD3E* rank ≤ 50, light when *CD3E* rank > 50).

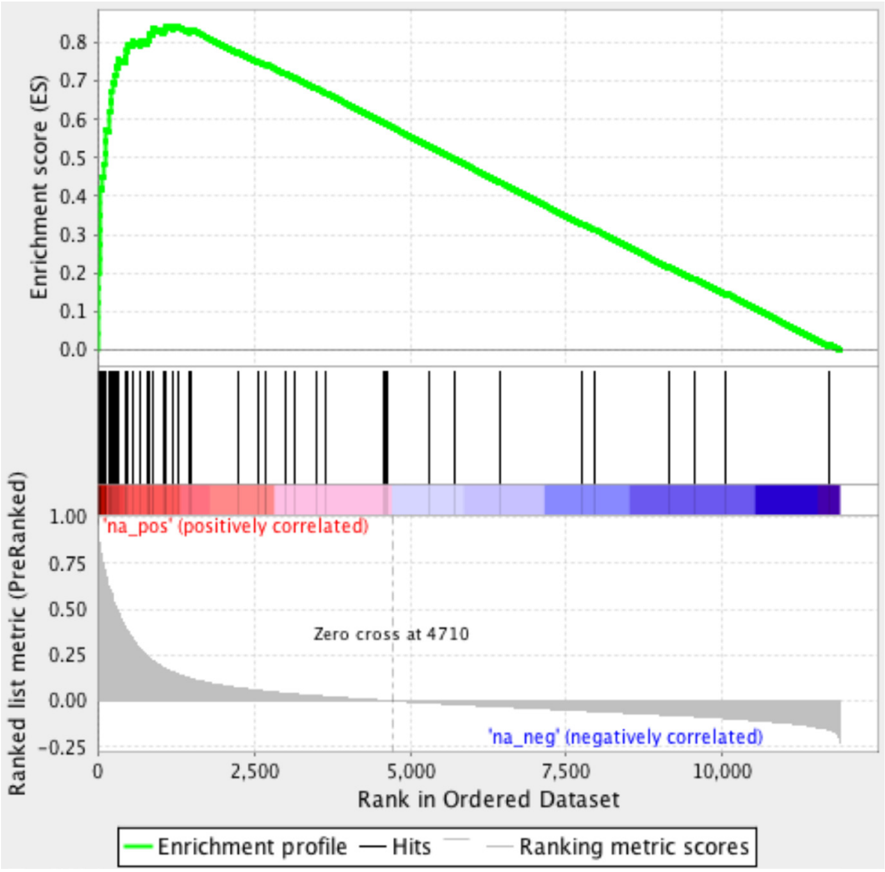

Supplementary Figure 5: GSEA summary plots for the pathway with highest enrichment when ranking by *CD3E* co-expression: TCR signaling in naïve CD4⁺ T cells, from the Pathway Interaction Database. The top plot profiles the enrichment score for the set of genes from the top-ranked gene to the index gene as the ranked gene list is traversed. The middle plot indicates the positions of genes in the TCR signaling in naïve CD4+ T cells pathway within the ranked list. The bottom plot profiles the co-expression values used to rank the gene list.

Supplementary Table 1: Top 50 enriched pathways based on gene set enrichment analysis

Rank	NESa	Pathway name
1	2.383	TCR pathway (PID)
2	2.366	CD8 TCR pathway (PID)
3	2.360	Immunoregulatory interactions between lymphoid and non-lymphoid cells (REACTOME)
4	2.315	Hematopoietic cell lineage (KEGG)
5	2.302	Class A1 rhodopsin like receptors (REACTOME)
6	2.302	Interferon gamma signaling (REACTOME)
7	2.300	Natural killer cell mediated cytotoxicity (KEGG)
8	2.280	Cell adhesion molecules cams (KEGG)
9	2.259	Cytokine cytokine receptor interaction (KEGG)
10	2.251	Costimulation by the CD28 family (REACTOME)
11	2.241	IL12 2pathway (PID)
12	2.235	Gpcr ligand binding (REACTOME)
13	2.233	Interferon alpha beta signaling (REACTOME)
14	2.226	Primary immunodeficiency (KEGG)
15	2.220	T cell receptor signaling pathway (KEGG)
16	2.219	TCR pathway (BIOCARTA)
17	2.218	G alpha i signalling events (REACTOME)
18	2.211	CD8 TCR downstream pathway (PID)
19	2.210	Bcr 5pathway (PID)
20	2.199	Leishmania infection (KEGG)
21	2.198	Innate immune system (REACTOME)
22	2.198	Chemokine signaling pathway (KEGG)
23	2.185	Generation of second messenger molecules (REACTOME)
24	2.184	T cell signal transduction (ST)
25	2.182	Peptide ligand binding receptors (REACTOME)
26	2.180	CXCR4 pathway (PID)
27	2.180	B cell receptor signaling pathway (KEGG)
28	2.174	Systemic lupus erythematosus (KEGG)
29	2.174	Antigen processing and presentation (KEGG)
30	2.170	PI3KCI pathway (PID)
31	2.170	Gpvi mediated activation cascade (REACTOME)
32	2.168	TCR signaling (REACTOME)
33	2.149	Toll like receptor signaling pathway (KEGG)
34	2.139	Gpcr downstream signaling (REACTOME)
35	2.125	IL12 STAT4 pathway (PID)
36	2.107	Cytokine signaling in immune system (REACTOME)
37	2.102	Interferon signaling (REACTOME)
38	2.088	Signaling by ils (REACTOME)
39	2.081	IL 3 5 and GM CSF signaling (REACTOME)
40	2.081	IL4 2pathway (PID)
41	2.079	Cell surface interactions at the vascular wall (REACTOME)
42	2.077	Nucleotide binding domain leucine rich repeat containing receptor NLR signaling pathways (REACTOME)

43	2.075	Antigen activates B cell receptor leading to generation of second messengers (REACTOME)
44	2.075	Intestinal immune network for IGA production (KEGG)
45	2.073	TXA2 pathway (PID)
46	2.073	Nkt pathway (BIOCARTA)
47	2.069	Leukocyte transendothelial migration (KEGG)
48	2.063	FCER1 pathway (PID)
49	2.061	IL8 CXCR2 pathway (PID)
50	2.058	Chemokine receptors bind chemokines (REACTOME)

[*] Normalized Enrichment Score (NES) is shown for each enriched pathways. All pathways listed had FDR $Q < 1 \times 10^{5}$.

Supplementary Table 2: Cancer type abbreviations and sample counts

Abbreviation	Cancer type	Sample count
ACC	Adrenocortical carcinoma	79
BLCA	Invasive Urothelial Bladder Cancer	408
BRCA	Breast Invasive Carcinoma	1100
CESC	Cervical squamous cell carcinoma & endocervical adenocarcinoma	306
CHOL	Cholangiocarcinoma	36
COADREAD	Colorectal Adenocarcinoma	382
DLBC	Lymphoid Neoplasm Diffuse Large B-cell Lymphoma	48
ESCA	Esophageal carcinoma	185
GBM	Glioblastoma Multiforme	166
HNSC	Head and Neck Squamous Cell Carcinoma	522
KICH	Chromophobe Renal Cell Carcinoma	66
KIRC	Clear Cell Carcinoma	534
KIRP	Kidney Papillary Carcinoma	291
LAML	Acute Myeloid Leukemia	173
LGG	Lower Grade Glioma	530
LIHC	Liver hepatocellular carcinoma	373
LUAD	Lung Adenocarcinoma	517
LUSC	Lung Squamous Cell Carcinoma	501
MESO	Mesothelioma	87
OV	Ovarian Serous Cystadenocarcinoma	307
PAAD	Pancreatic adenocarcinoma	179
PCPG	Pheochromocytoma and Paraganglioma	184
PRAD	Prostate Adenocarcinoma	498
SARC	Sarcoma	263
SKCM	Cutaneous Melanoma	472
STAD	Stomach Adenocarcinoma	415
TGCT	Testicular Germ Cell Cancer	156
THCA	Thyroid Carcinoma	509
UCEC	Uterine Corpus Endometrial Carcinoma	177
UCS	Uterine Carcinosarcoma	57
UVM	Uveal Melanoma	80
